# Impact of beta blockers on patients undergoing transcatheter aortic valve replacement: the OCEAN-TAVI registry

**DOI:** 10.1136/openhrt-2020-001269

**Published:** 2020-07-07

**Authors:** Tetsuya Saito, Nobuhiro Yoshijima, Hiromu Hase, Fumiaki Yashima, Hikaru Tsuruta, Hideyuki Shimizu, Keiichi Fukuda, Toru Naganuma, Kazuki Mizutani, Motoharu Araki, Norio Tada, Futoshi Yamanaka, Shinichi Shirai, Minoru Tabata, Hiroshi Ueno, Kensuke Takagi, Akihiro Higashimori, Yusuke Watanabe, Masanori Yamamoto, Kentaro Hayashida

**Affiliations:** 1 Cardiology, Keio University School of Medicine, Shinjuku-ku, Tokyo, Japan; 2 Cardiology, Saiseikai Utsunomiya Hospital, Utsunomiya, Tochigi, Japan; 3 Cardiovascular Surgery, Keio University School of Medicine, Shinjuku-ku, Tokyo, Japan; 4 Cardiology, New Tokyo Hospital, Matsudo, Chiba, Japan; 5 Cardiovascular Medicine, Osaka City General Hospital, Osaka, Osaka, Japan; 6 Cardiology, Saiseikai Yokohama-City Eastern Hospital, Yokohama, Kanagawa, Japan; 7 Cardiology, Sendai Kosei Hospital, Sendai, Miyagi, Japan; 8 Cardiology, Shonan Kamakura General Hospital, Kamakura, Kanagawa, Japan; 9 Cardiology, Kokura Memorial Hospital, Kitakyushu, Fukuoka, Japan; 10 Cardiovascular Surgery, Tokyo Bay Urayasu Ichikawa Iryo Center, Urayasu, Chiba, Japan; 11 Cardiology, Toyama University School of Medicine, Toyama, Toyama, Japan; 12 Cardiology, Ogaki Municipal Hospital, Ogaki, Gifu, Japan; 13 Cardiology, Kishiwada Tokushukai Hospital, Kishiwada, Osaka, Japan; 14 Cardiology, Teikyo University School of Medicine, Itabashi-ku, Tokyo, Japan; 15 Cardiology, Toyohashi Heart Center, Toyohashi, Aichi, Japan; 16 Cardiology, Nagoya Heart Center, Nagoya, Aichi, Japan

**Keywords:** aortic valve disease, beta blockers, percutaneous valve therapy

## Abstract

**Objective:**

There is paucity of data on optimal medical treatment, including use of beta blockers for patients undergoing transcatheter aortic valve replacement (TAVR). The study aimed to investigate the association of beta blockers and clinical outcomes following TAVR.

**Methods:**

We examined data of 2563 patients who underwent TAVR between October 2013 and May 2017 obtained from a prospective multicentre cohort registry, the optimised catheter valvular intervention-TAVI registry. We compared the 2-year cardiovascular and non-cardiovascular mortality and in-hospital outcomes between patients with and without preprocedural beta-blocker administration by propensity score matching (PSM).

**Results:**

Preprocedural beta blockers were prescribed in 867 patients (33.8%). After PSM, the incidence of in-hospital congestive heart failure was significantly lower in patients with preprocedural beta blocker (p=0.046). No differences were found in 2-year cardiovascular and non-cardiovascular mortality. In the subgroup analyses, beta-blocker administration was associated with a lower cardiovascular mortality within 2 years in patients with a history of coronary artery bypass grafting (CABG; log-rank p=0.017), presence of peripheral artery disease (PAD; log-rank p=0.003) and brain natriuretic peptide (BNP) ≥400 pg/mL (log-rank p=0.003). When stratified by postprocedural left ventricular ejection fraction (post-LVEF), beta-blocker administration was associated with a lower cardiovascular mortality among patients with post-LVEF <50% (log-rank p=0.024).

**Conclusions:**

Preprocedural beta-blocker administration was not associated with 2-year cardiovascular and non-cardiovascular mortality in overall, but was associated with a lower 2-year cardiovascular mortality in patients with a history of CABG, presence of PAD, BNP ≥400 pg/mL and post-LVEF <50%. The findings must be validated using randomised trials.

**Key questions**

**What is already known about this subject?**

- Transcatheter aortic valve replacement (TAVR) is an established therapy for symptomatic patients with severe aortic stenosis. However, the optimal medical therapy, including beta blockers for patients who undergo TAVR, remains unspecified.

**What does this study add?**

- The study showed that beta-blocker administration was associated with a lower 2-year cardiovascular mortality in patients with a history of coronary artery bypass grafting (CABG; log-rank p=0.017), presence of peripheral artery disease (PAD; log-rank p=0.003), brain natriuretic peptide (BNP) ≥400 pg/mL and postprocedural left ventricular ejection fraction (LVEF) <50% (log-rank p=0.024).

**How might this impact on clinical impact?**

- This result suggests that beta-blocker administration was associated with good clinical outcomes among patients with a history of CABG, presence of PAD, BNP ≥400 pg/mL and post-procedural LVEF <50%. The findings need to be confirmed by randomised trials.

## Introduction

It is not clear whether medical therapy is advantageous for the treatment of aortic stenosis (AS). Several prior studies have shown that renin–angiotensin system (RAS) inhibitors for AS are associated with improved survival.^1 2^ It is also well known that beta blockers should be carefully administered in cases of severe AS because of their negative inotropic effects. However, there are conflicting reports on survival benefits when beta blockers are used in patients with severe AS.^3^ Beta blockers reduce the haemodynamic and metabolic overload in patients with asymptomatic severe AS.^4^

Transcatheter aortic valve replacement (TAVR) is an established therapy in patients with symptomatic severe AS at high or intermediate surgical risk.^5–7^ Recently, the placement of aortic transcatheter valves 3 trial showed that TAVR with a balloon-expandable valve was more effective than surgical aortic valve replacement (SAVR) with respect to the composite endpoint of death, stroke and rehospitalisation at 1 year in patients with severe AS at low surgical risk.^8^ Besides, TAVR with a self-expanding valve was non-inferior to SAVR with respect to death or disabling stroke in patients at low surgical risk.^9^ Although the indication of TAVR is expanding based on these studies, there are limited data regarding the optimal medical treatment for patients undergoing TAVR. The use of RAS inhibitors after TAVR has lowered mortality.^10 11^ However, the effect of preprocedural beta-blocker administration on these patients remains unclear. Thus, our purpose was to investigate the effect of preprocedural administration of beta blockers on the clinical outcomes of patients with severe AS who underwent TAVR.

## Methods

### Study population

A total of 2588 patients were enrolled in the Optimised transCathEter vAlvular iNtervention (OCEAN)-transcatheter aortic valve implantation (TAVI) registry between October 2013 and May 2017. The OCEAN-TAVI registry is a prospective, multicentre, observational registry of patients who underwent TAVR using the Edwards Sapien XT/Sapien 3 prosthesis (Edwards Lifesciences, Irvine, California, USA) or Medtronic Corevalve/Evolut R prosthesis (Medtronic, Minneapolis, Minnesota, USA) at 14 Japanese medical centres. All study participants provided informed consent and the registry was approved by the ethics committees of all participating institutions. Patients or the public were not involved in the design, or conduct, or reporting, or dissemination plans of our research. The OCEAN-TAVI registry was registered with the University Hospital Medical Information Network Clinical Trial Registry and accepted by the International Committee of Medical Journal Editors (UMIN-ID: 000020423). Patients were followed annually at the participating institutions. At that time, blood tests, echocardiography were performed. The events were site reported from the participating institutions and not adjudicated in clinical event adjudication committee. The database was regularly audited by the data committee members to ensure the consistency.

**Figure 1:**
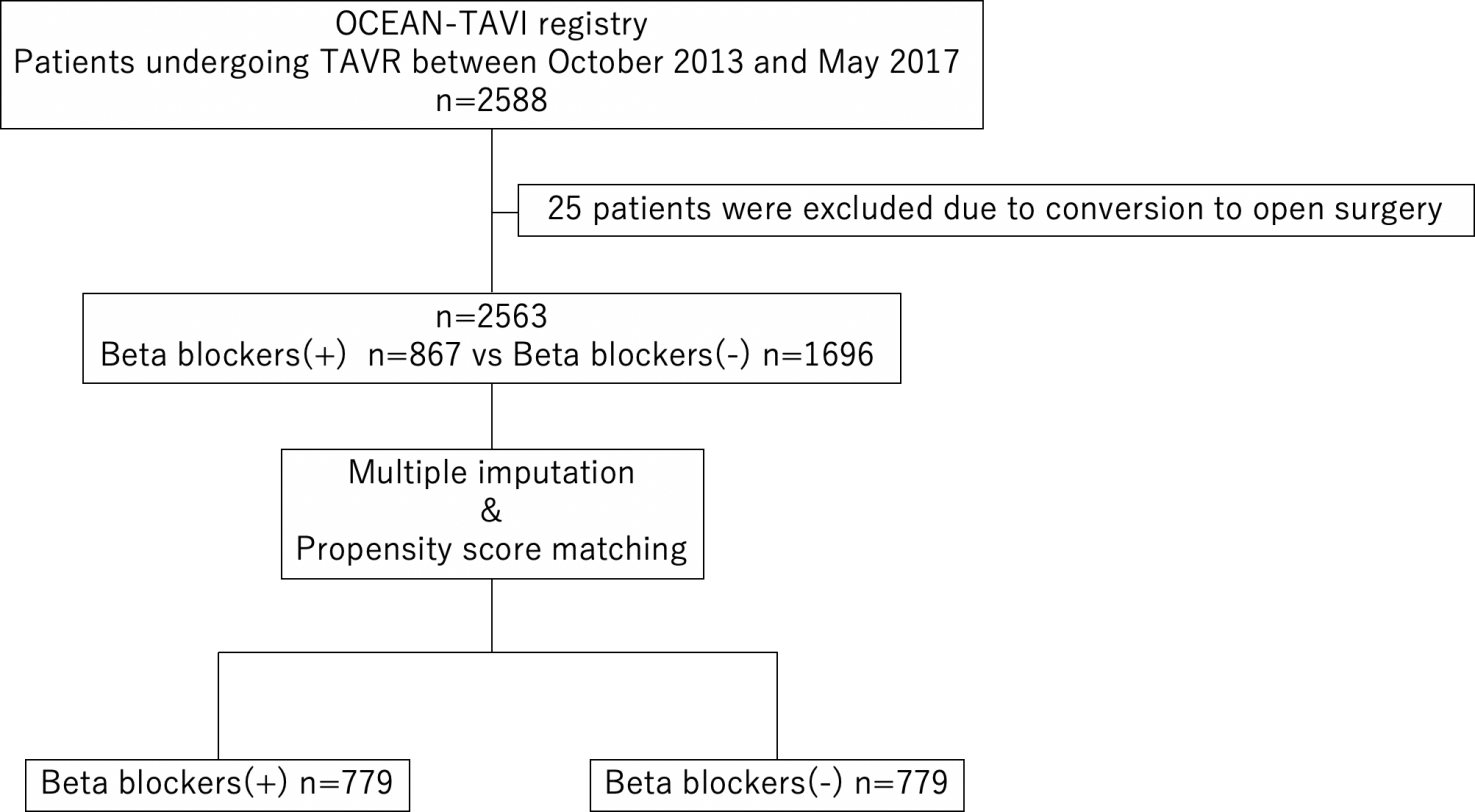
Study flow diagram. Of 2588 patients, 25 patients were excluded due to conversion to open surgery. Beta-blocker (−)=patients without preprocedural beta blockers; beta-blocker (+)=patients with preprocedural beta blockers. OCEAN, Optimised transCathEter vAlvular iNtervention; TAVR, transcatheter aortic valve replacement.

After exclusion of 25 patients owing to conversion to open surgery, 2563 patients participated in this study and were divided based on whether or not they were administered preprocedural beta blockers (figure 1). Preprocedural beta-blocker administration was defined as beta blockers being prescribed at TAVR.

### Outcomes

The primary outcomes were 2-year cardiovascular and non-cardiovascular mortalities after TAVR. For patients who lost to follow-up, we used the last date when survival was confirmed. The secondary outcomes were as follows: in-hospital outcomes and complications and postprocedural echocardiographic data. Cardiovascular mortality and complications were defined based on the Valve Academic Research Consortium (VARC)-2 criteria.^12^ In-hospital congestive heart failure was defined as the requirement of intravenous injection of diuretics or inotropic agents, mechanical support for heart failure, such as intra-aortic balloon pumping (IABP) or extracorporeal membrane oxygenation (ECMO) after TAVR.

### Echocardiography

Transthoracic echocardiography was performed at baseline, before hospital discharge, and at the annual follow-up. All transthoracic echocardiographic parameters were measured according to the guidelines of the American Society of Echocardiography.^13 14^ The degree of paravalvular leak (PVL) was measured in accordance with the VARC-2 criteria and reported as a semiquantitative grade: none, trace, mild, moderate and severe. Prosthesis-patient mismatch (PPM) was defined as an indexed effective orifice area <0.85 cm^2^/m^2^ according to the VARC-2 criteria.

### Statistical analysis

We compared baseline characteristics between patients with and without preprocedural administration of beta blockers (table 1). Continuous variables are presented as medians and IQRs (25%–75%) and compared using the Student’s t-test or Mann-Whitney U test. Categorical variables are presented as means and percentages and compared using the Pearson’s χ^2^ test or Fisher’s exact test.

**Table 1:** Baseline characteristics

	Beta-blocker (+) n=867	Beta-blocker (−) n=1696	P value	SMD
Age, years	85(82–88)	85(81–88)	0.91	0.005
Male	262 (30.2)	527 (31.1)	0.66	0.019
Body mass index, kg/m^2^	21.9 (19.4–24.4)	22.1 (19.7–24.3)	0.44	0.032
Body surface area, m^2^	1.40 (1.30–1.52)	1.41 (1.30–1.54)	0.28	0.045
NYHA 3 or 4	479 (55.2)	827 (48.8)	0.002	0.13
Hypertension	691 (79.7)	1280 (75.5)	0.016	0.1
Dyslipidaemia	375 (43.3)	729 (43.0)	0.896	0.005
Diabetes mellitus	196 (22.6)	351 (20.7)	0.26	0.046
Chronic kidney disease	645 (74.4)	1146 (67.6)	<0.001	0.15
Previous ischaemic stroke	78 (9.0)	205 (12.1)	0.018	0.1
Previous haemorrhagic stroke	3 (0.3)	9 (0.5)	0.52	0.028
COPD	138 (15.9)	243 (14.3)	0.29	0.044
Peripheral artery disease	145 (16.7)	227 (13.4)	0.023	0.093
Coronary artery disease	349 (40.3)	597 (35.2)	0.012	0.1
Previous CABG	71 (8.2)	98 (5.8)	0.02	0.095
Atrial fibrillation	227 (26.2)	316 (18.6)	<0.001	0.18
Permanent pacemaker	70 (8.1)	96 (5.7)	0.019	0.096
Liver disease	27 (3.1)	49 (2.9)	0.75	0.013
Active cancer	43 (5.0)	81 (4.8)	0.84	0.009
Clinical frail score			0.36	0.060
1–4	655 (75.5)	1237 (72.9)		
5,6	180 (23.1)	392 (23.1)		
7,8	32 (3.7)	67 (4.0)		
STS score, %	6.96 (4.84–10.2)	6.30 (4.35–9.24)	<0.001	0.063
Logistic EuroScore, %	13.6 (8.83–22.1)	12.7 (8.10–20.5)	0.011	0.069
Euro II score, %	4.06 (2.61–6.30)	3.57 (2.24–5.87)	0.001	0.037
Medication				
RAS inhibitors	484 (55.8)	889 (52.4)	0.1	0.068
Ca blockers	376 (43.4)	749 (44.2)	0.7	0.016
Digoxin	22 (2.5)	66 (3.9)	0.075	0.077
Any diuretic therapy	570 (65.7)	801 (47.2)	<0.001	0.38
Statin	380 (43.8)	678 (40.0)	0.061	0.078
Laboratory				
Na, mEq/L	140 (138–142)	140 (138–142)	0.55	0.002
Hb, g/L	110 (100–123)	113 (102–125)	0.022	0.081
eGFR, mL/min/1.73 m^2^	47.6 (35.1–60.1)	51.7 (39.0–64.1)	<0.001	0.2
Albumin, g/dL	3.80 (3.40–4.00)	3.80 (3.50–4.10)	0.005	0.11
Albumin <3.5 g/dL	223 (25.7)	383 (22.6)	0.077	0.073
Brain natriuretic peptide, pg/mL	355 (169–666)	231 (103–515)	<0.001	0.18
Brain natriuretic peptide ≥ 400 pg/mL	322 (45.2)	499 (32.8)	<0.001	0.26
Preprocedural echocardiographic data/computed tomographic data				
Aortic valve are, cm^2^	0.61 (0.50–0.73)	0.63 (0.51–0.75)	0.033	0.086
Peak velocity, m/s	4.40 (4.00–4.98)	4.58 (4.10–5.12)	<0.001	0.22
Mean pressure gradient, mm Hg	45.5 (36.0–57.7)	49.0 (39.0–63.0)	<0.001	0.22
LV end-diastolic diameter, mm	44.0 (39.5–49.0)	43.8 (40.0–48.0)	0.88	0.035
LV end-systolic diameter, mm	28.3 (25.0–34.1)	28.0 (24.5–32.4)	0.012	0.12
Left atrial diameter, mm	43.0 (37.9–47.0)	41.1 (37.1–46.0)	0.004	0.089
LVEF, %	61.0 (51.0–66.9)	63.0 (53.1–68.1)	<0.001	0.15
LVEF <50%	201 (23.2)	324 (19.1)	0.016	0.099
Indexed stroke volume, mL/m^2^	42.5 (33.2–52.7)	45.4 (36.9–54.4)	<0.001	0.22
Indexed stroke volume <35 mL/m^2^	213 (29.6)	281 (20.2)	<0.001	0.22
AR ≥moderate	98 (11.3)	176 (10.4)	0.48	0.03
MR ≥moderate	118 (13.6)	170 (10.0)	0.007	0.11
Bicuspid	23 (2.7)	41 (2.4)	0.72	0.015
Annulus area, mm^2^	389 (346–442)	390 (349–439)	0.75	0.01
SOV mean diameter, mm	29.3 (27.6–31.4)	29.4 (27.6–31.5)	0.4	0.024
Procedural data				
Transfemoral approach	700 (80.7)	1446 (85.3)	0.003	0.12
Elective	810 (93.4)	1604 (94.6)	0.95	0.048
Local anaesthesia	206 (23.8)	401 (23.6)	0.65	0.003

AR, aortic regurgitation; CABG, coronary artery bypass grafting; COPD, chronic obstructive pulmonary disease; eGFR, estimated glomerular filtration rate; Hb, haemoglobin; LV, left ventricular; LVEF, left ventricular ejection fraction; MR, mitral regurgitation; NYHA, New York Heart Association; RAS, renin–angiotensin system; SMD, standardised mean difference; SOV, sinus of Valsalva; STS, Society of Thoracic Surgeons Predicted Risk of Mortality.

Baseline variables are listed in table 1. There were missing data for baseline variables. Percentage of missing data for baseline variables are shown in online supplementary table 1. There were no missing data for baseline variables not in online supplementary table 1. Multiple imputation was performed to properly handle missing data. Missing continuous variables were imputed using the predictive mean matching method. Missing binary variables were imputed using logistic regression models. We created 20 imputed datasets. Propensity score matching (PSM) was performed after multiple imputations to reduce imbalances at baseline between the groups. The covariates included in the model are listed in table 1. Propensity scores were calculated within each imputed dataset using logistic regression models to estimate the probability of preprocedural beta-blocker prescription. Propensity scores were averaged across the imputed datasets for each patient. PSM was performed with a 1:1 matching protocol without replacement by the averaged propensity scores. The calliper width was 0.2 of the SD of the logit of the averaged propensity scores. Balance between the two groups was assessed by absolute standardised mean difference (SMD).

10.1136/openhrt-2020-001269.supp1 Supplementary data

Cumulative incidences were calculated using the Kaplan-Meier method in the matched cohort. The log-rank test was performed to compare cardiovascular mortality between patients with and without preprocedural administration of beta blockers.

To identify the groups for which beta blockers were effective, subgroup analyses for cardiovascular mortality were performed for age (≥80 or <80 years), sex, chronic kidney disease (CKD), atrial fibrillation (AF), coronary artery disease (CAD), coronary artery bypass grafting (CABG), peripheral artery disease (PAD), preprocedural left ventricular ejection fraction (pre-LVEF; ≥50% or <50%), preprocedural aortic regurgitation (AR; ≥moderate or ≤mild), preprocedural mitral regurgitation (MR; ≥moderate or ≤mild) and brain natriuretic peptide (BNP; ≥400 pg/mL or <400 pg/mL). In subgroup analyses for cardiovascular mortality, patients with BNP ≥400 pg/mL were focused because it is said that there is a high possibility of heart failure if BNP ≥400 pg/mL.^15 16^ In addition, we performed the subgroup analysis for cardiovascular mortality in the subset of PPM and PVL.

To confirm the safety of beta blockers for patients undergoing TAVR, subgroup analyses for non-cardiovascular mortality were performed for age (≥80 or <80 years), sex, body mass index (≥20 kg/m^2^ or <20 kg/m^2^), New York Heart Association (NYHA) class (1, 2 or 3, 4), CKD, PAD, chronic obstructive pulmonary disease (COPD), albumin levels (≥3.5 g/dL or <3.5 g/dL) and clinical frail score (1–4, 5–6 or ≥7). Interaction tests between each covariate were performed. PSM after multiple imputation was performed in the subgroups with p for interaction <0.05. We selected the subgroup from the original data including 2563 patients, then performed PSM for preprocedural beta blockers because the baseline characteristic between beta-blocker group and non-beta-blocker group would be consistent in the subgroup. The log-rank test was performed to evaluate cardiovascular mortality between patients with and without preprocedural beta-blocker administration in the subgroups.

It was hypothesised that the effect of beta blockers on cardiovascular mortality would be influenced by LVEF. Therefore, the original data were divided according to pre-LVEF (≥50% or <50%), the postprocedural LVEF (post-LVEF; ≥50% or <50%). PSM for preprocedural beta-blocker administration was performed in each group. The log-rank test was performed to evaluate cardiovascular mortality.

All statistical analyses were performed using SPSS V.23.0 (IBM SPSS Statistics, IBM Corporation) and R software V.3.5.2 (Vienna, Austria). All tests were two sided and statistical significance was set at p<0.05.

## Results

### Baseline characteristics

The baseline characteristics of the study participants are shown in table 1. Among 2563 patients, beta blockers were administered to 867 patients (33.8%) before TAVR despite the presence of severe AS (figure 1). There were statistically significant differences between patients with and without beta-blocker administration with regard to NYHA class 3 or 4, history of ischaemic stroke, CABG, presence of hypertension, CKD, PAD, CAD, AF, implantation of permanent pacemaker, Society of Thoracic Surgeons Predicted Risk of Mortality score, Logistic Euro score, Euro II score, any diuretic therapy, haemoglobin, estimated glomerular filtration rate, albumin levels, BNP, aortic valve area, peak velocity, mean pressure gradient, LV end-systolic diameter, left atrial diameter, LVEF, indexed stroke volume, MR ≥moderate and the transfemoral approach.

### Primary outcomes in the matched cohort

A total of 1558 patients were matched (online supplementary table 2). After performing PSM, the absolute SMD value was lower than 0.1 in all examined covariates.

**Figure 2:**
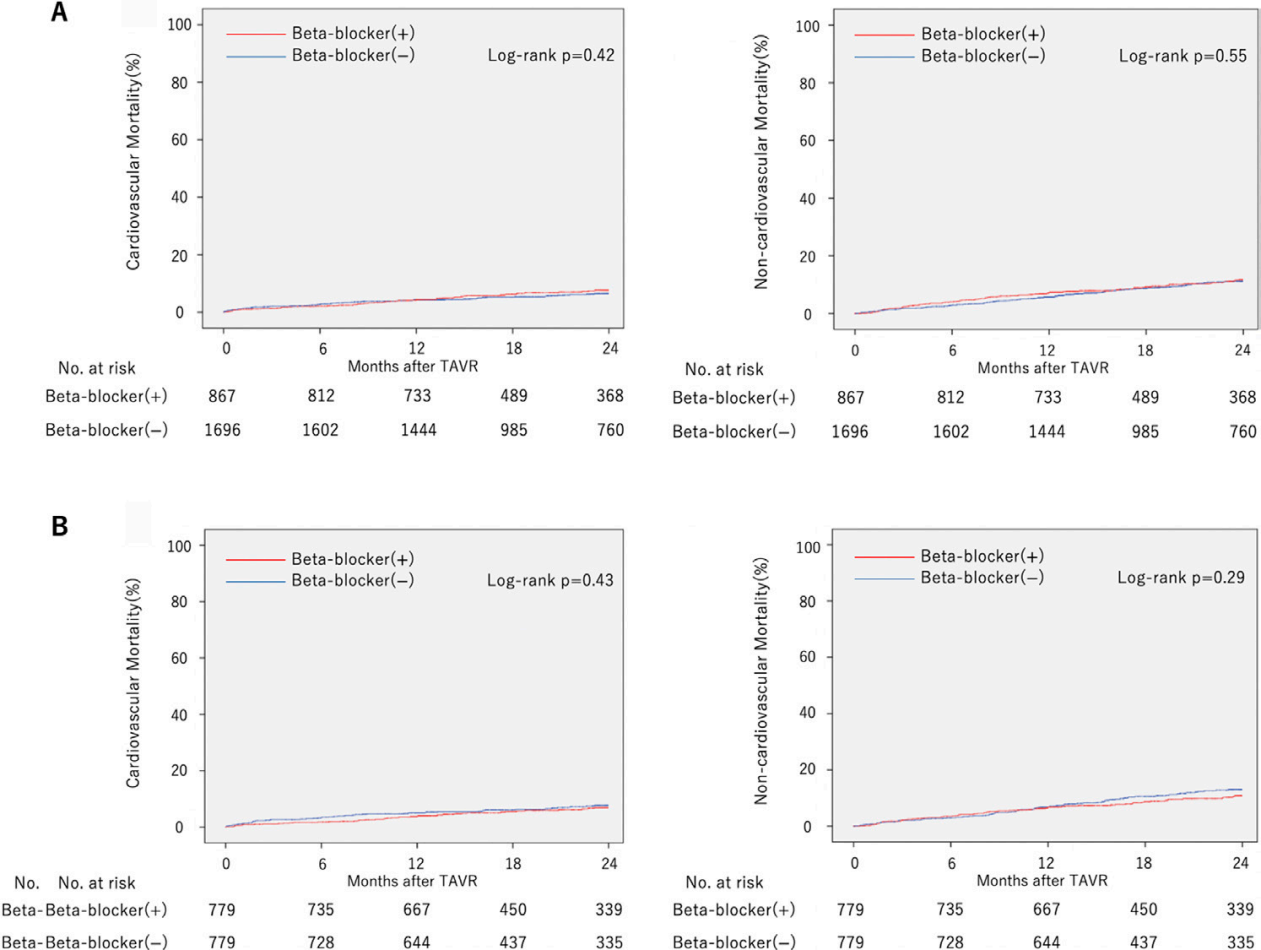
Kaplan-Meier curves of cardiovascular mortality and non-cardiovascular mortality in the non-matched and matched cohort. Two-year cardiovascular mortality and non-cardiovascular mortality of patients with beta-blocker administration compared with those without in the (A) non-matched and (B) matched cohort. TAVR, transcatheter aortic valve replacement.

Kaplan-Meier curves of cardiovascular and non-cardiovascular mortality in the non-matched and matched cohort are shown in figure 2. Follow-up rate at 2 years was 92.1%. The median follow-up period in the matched cohort was 658 days (IQR 381–863). Ninety-five patients died of cardiovascular causes and 154 of non-cardiovascular causes during the follow-up period in the matched cohort. There was no significant difference between the two groups regarding cardiovascular (log-rank p=0.43) and non-cardiovascular mortality (log-rank p=0.29) in the matched cohort.

### Secondary outcomes in the matched cohort

Regarding the matched cohort, in-hospital patient outcomes, complications and postprocedural echocardiographic data are shown in table 2. The incidence of in-hospital congestive heart failure was lower among patients treated with beta blockers than among those treated without (p=0.046).

**Table 2:** In-hospital outcomes and postprocedural echocardiographic data

	Beta-blocker (+) n=779	Beta-blocker (−) n=779	P value
Procedural outcomes			
30-day mortality	8 (1.0)	14 (1.8)	0.19
Procedural MI	6 (0.8)	1 (0.1)	0.12
Ischaemic stroke	13 (1.7)	15 (1.9)	0.7
Haemorrhagic stroke	1 (0.1)	3 (0.4)	0.32
Bleeding	190 (24.4)	192 (24.6)	0.91
AKI	91 (11.7)	93 (11.9)	0.88
Vascular complication	74 (9.5)	68 (8.7)	0.59
New permanent pacemaker	56 (7.2)	74 (9.5)	0.099
New onset AF	34 (4.4)	24 (3.1)	0.19
Congestive heart failure	34 (4.4)	52 (6.7)	0.046
Postprocedural echocardiographic data			
LVEF, %	62.0 (54.0–67.0)	62.1 (54.0–67.4)	0.49
LVEF <50%	141 (18.3)	122 (15.9)	0.21
THV peak velocity, m/s	2.15 (1.90–2.47)	2.20 (1.93–2.50)	0.095
THV mean pressure gradient, mm Hg	9.4 (7.4–12.9)	10.0 (7.3–13.0)	0.13
Indexed EOA, cm^2^/m^2^	1.15 (0.97–1.36)	1.13 (0.95–1.31)	0.13
PPM	94 (12.4)	85 (11.3)	0.52
PVL			0.34
None	140 (18.0)	127 (16.4)	
Trace	366 (47.0)	377 (48.6)	
Mild	252 (32.4)	259 (33.4)	
Moderate	20 (2.6)	11 (1.4)	
Severe	1 (0.1)	0 (0.0)	
PVL ≥ moderate	20 (2.6)	12 (1.5)	0.16

AF, atrial fibrillation; AKI, acute kidney injury; EOA, effective orifice area; LVEF, left ventricular ejection fraction; MI, myocardial infarction; PPM, prosthesis-patient mismatch; PVL, paravalvular leak; THV, transcatheter heart valve.

### Subgroup analyses

**Figure 3:**
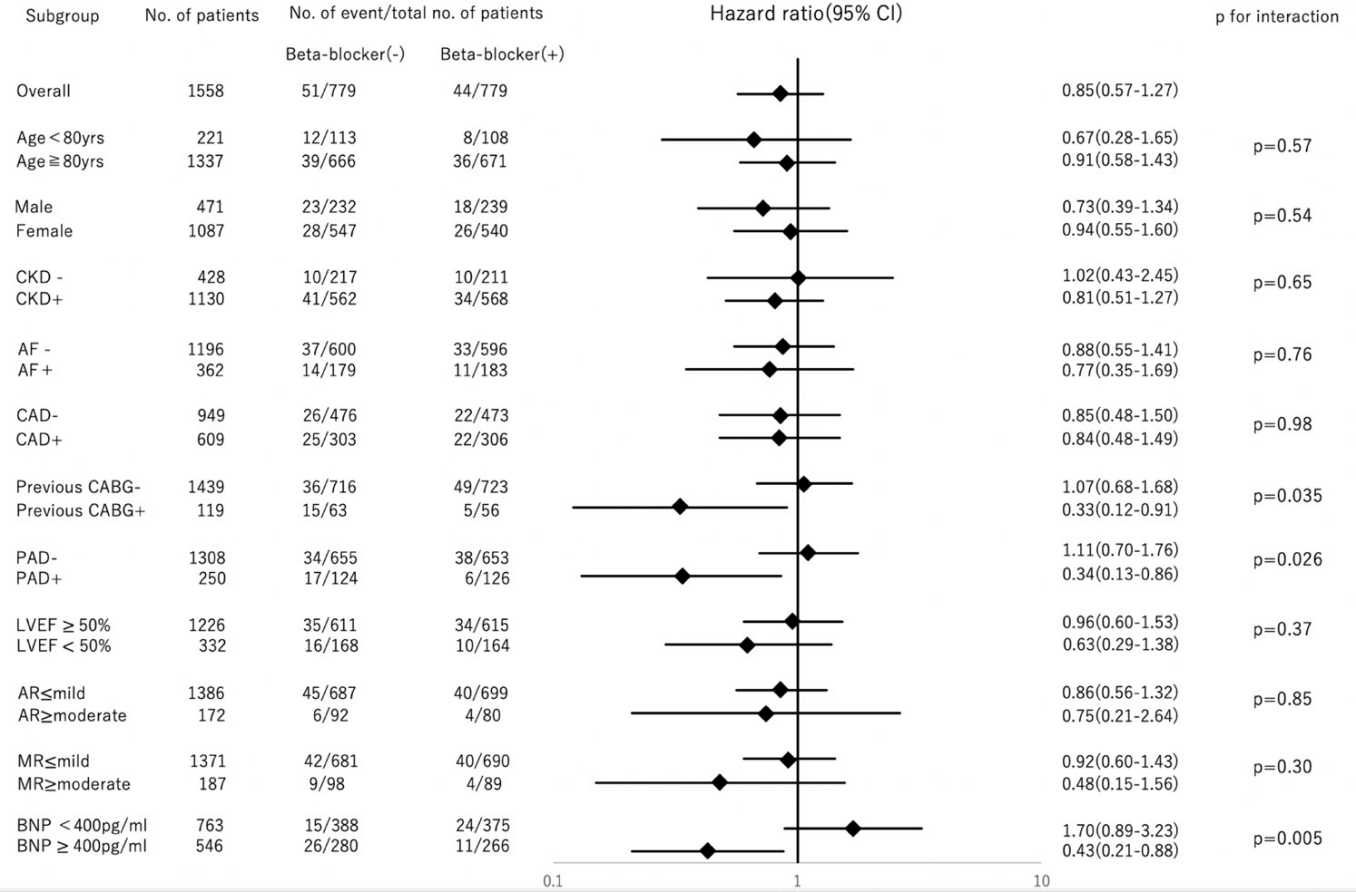
Subgroup analyses for cardiovascular mortality. Beta-blocker (−)=patients without preprocedural beta blockers; beta-blocker (+)=patients with preprocedural beta blockers. Forest plot representing the HRs of cardiovascular mortality in patients with beta-blocker administration compared with patients without, stratified by preprocedural characteristics. AF, atrial fibrillation; AR, aortic regurgitation; BNP, brain natriuretic peptide; CABG, coronary artery bypass grafting; CAD, coronary artery disease; CKD, chronic kidney disease; LVEF, left ventricular ejection fraction; MR, mitral regurgitation; PAD, peripheral artery disease.

**Figure 4:**
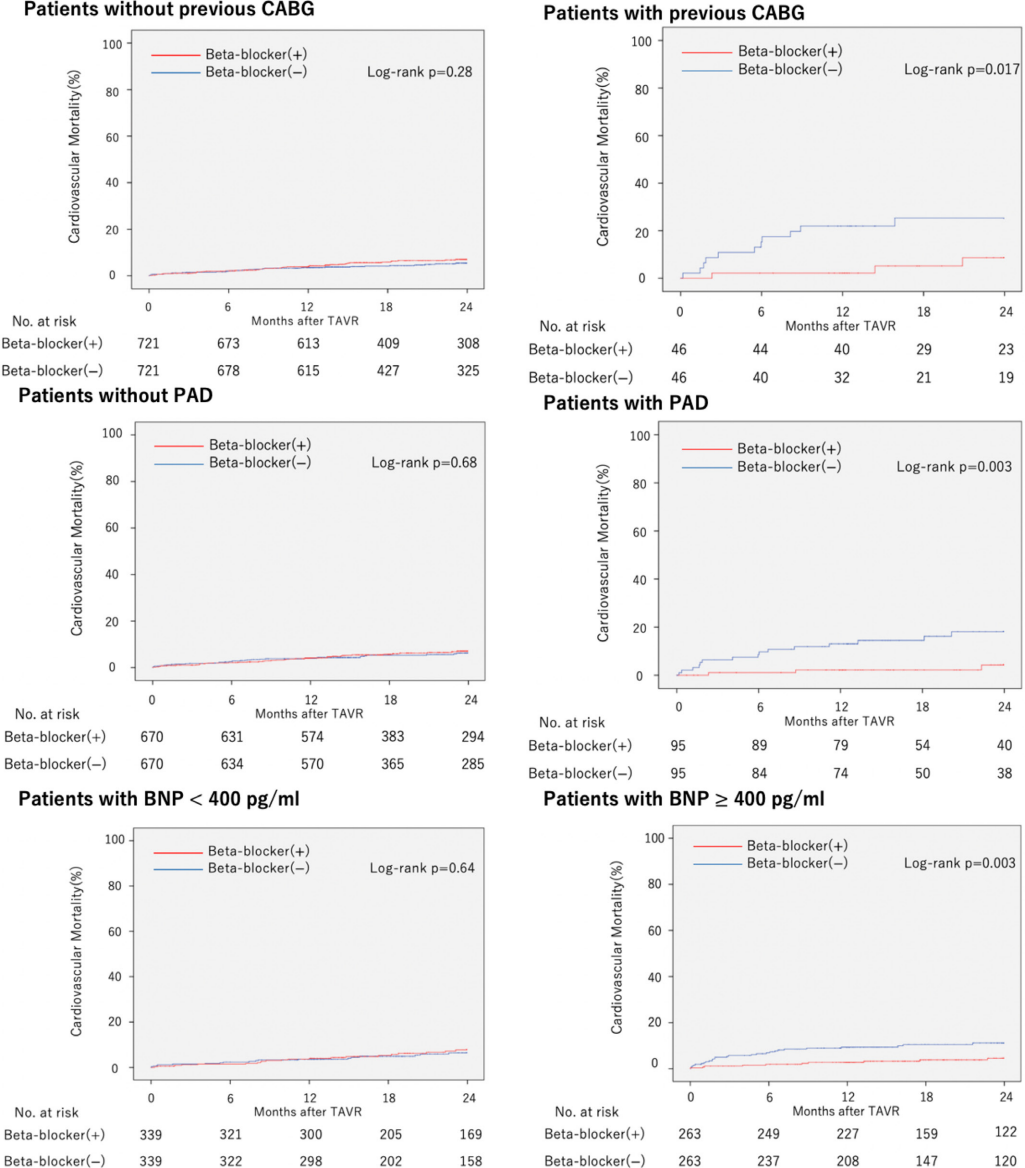
Cardiovascular mortality in subgroups. Kaplan-Meier curve of cardiovascular mortality in patient with and without a history of coronary artery bypass grafting (CABG), with and without presence of peripheral artery disease (PAD), with brain natriuretic peptide (BNP) <400 pg/mL and ≥400 pg/mL. TAVR, transcatheter aortic valve replacement.

Subgroup analyses for cardiovascular mortality are shown in figure 3. The groups with a significant difference were history of CABG (p for interaction=0.035), presence of PAD (p for interaction=0.026) and BNP (p for interaction=0.005). Based on this result, PSM was performed in the groups. Kaplan-Meier curves of cardiovascular mortality in each group are shown in figure 4. Beta-blocker administration was associated with a significantly lower 2-year cardiovascular mortality in patients with CABG history (log-rank p=0.017), presence of PAD (log-rank p=0.003) and BNP ≥400 pg/mL (log-rank p=0.003). Subgroup analyses for cardiovascular mortality in the subset of PPM and PVL are shown in online supplementary figure 1. There was no interaction between beta blockers and PPM (p for interaction=0.15), beta blocker and PVL (p for interaction=0.34).

**Figure 5:**
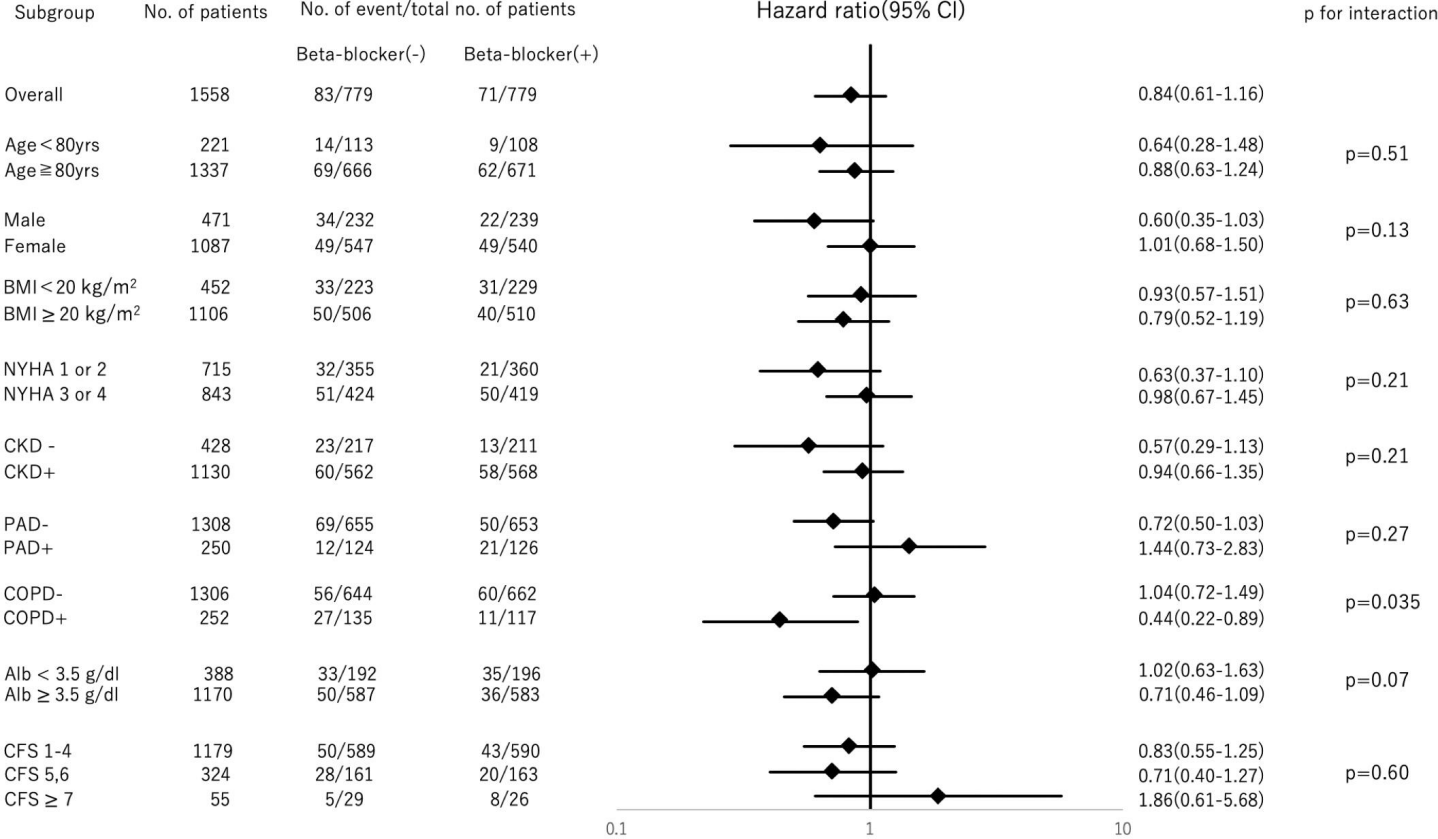
Subgroup analyses for non-cardiovascular mortality. Forest plot representing the HRs of non-cardiovascular mortality in patients with beta-blocker administration compared with patients without, stratified by preprocedural characteristics. BMI, body mass index; CFS, Clinical Frail Score; CKD, chronic kidney disease; COPD, chronic obstructive pulmonary disease; NYHA, New York Heart Association; PAD, peripheral artery disease.

Subgroup analyses for non-cardiovascular mortality are shown in figure 5. Interaction between COPD and beta-blocker administration was noted (p for interaction=0.035).

### Cardiovascular mortality stratified by LVEF

**Figure 6:**
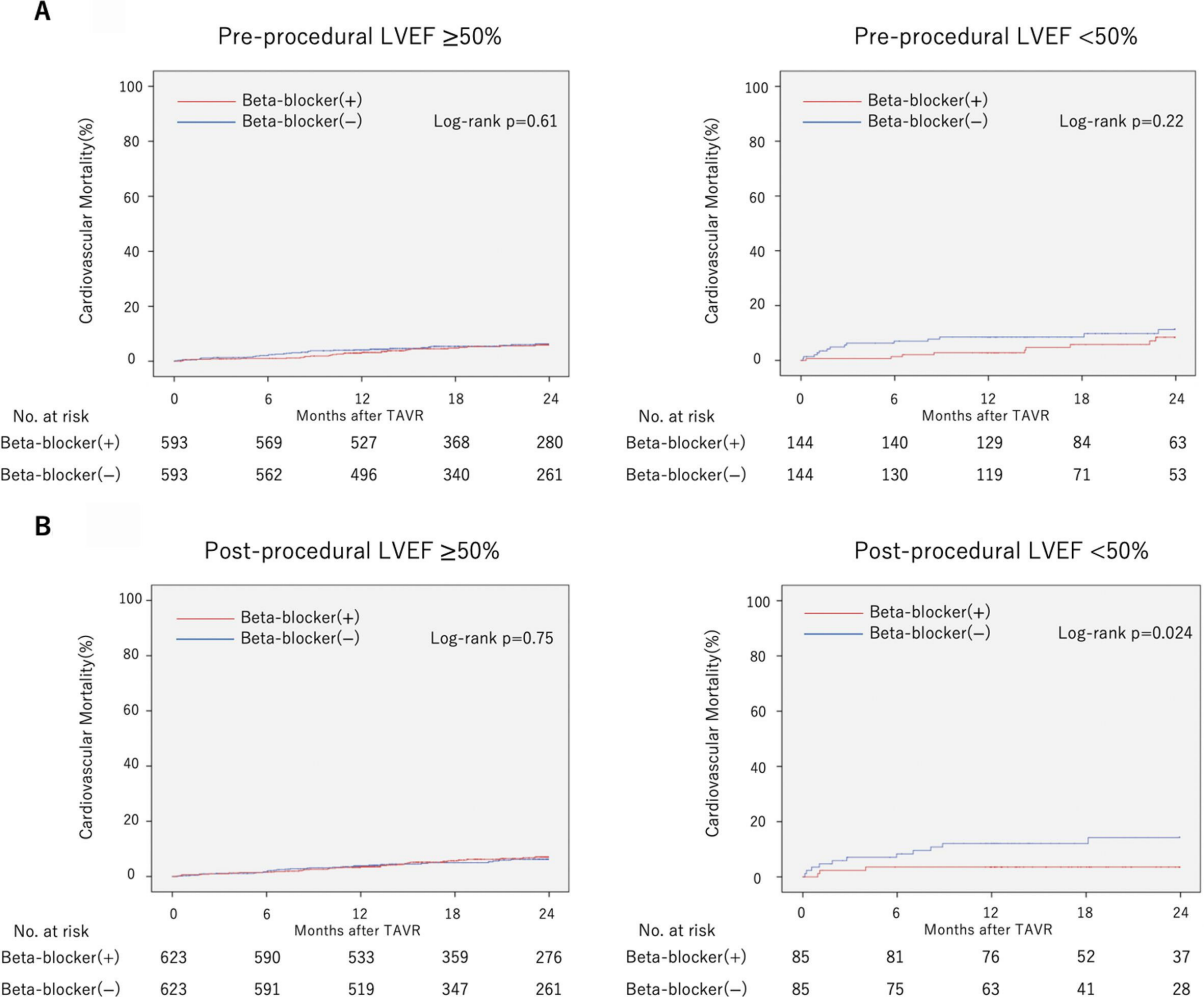
Cardiovascular mortality stratified by left ventricular ejection fraction (LVEF). Kaplan-Meier curve of cardiovascular mortality in patient with preprocedural LVEF ≥50% or <50% (A), postprocedural LVEF ≥50% or <50% (B). TAVR, transcatheter aortic valve replacement.

Kaplan-Meier curves of cardiovascular mortality in each group (pre-LVEF ≥50% or <50%, post-LVEF ≥50% or <50%) are shown in figure 6. The administration of beta blockers to patients with post-LVEF <50% was associated with a lower 2-year cardiovascular mortality (log-rank p=0.024). In patients with pre-LVEF ≥50%, pre-LVEF <50% and post-LVEF ≥50%, there was no significant difference in cardiovascular mortality between the groups with or without beta-blocker administration (log-rank p=0.61 in patients with pre-LVEF ≥50%, log-rank p=0.22 in patients with pre-LVEF <50% and log-rank p=0.75 in patients with post-LVEF ≥50%).

## Discussion

The optimal medical therapy for patients undergoing TAVR remains unknown. The main findings of our study showed that the administration of beta blockers to patients undergoing TAVR was associated with a lower incidence of in-hospital congestive heart failure and cardiovascular mortality in specific groups of patients (patients with a history of CABG, presence of PAD, BNP ≥400 pg/mL, and post-LVEF <50%). To our knowledge, the present study is the first to identify the association of beta blockers and clinical outcomes after TAVR.

Apart from some trial demonstrations of survival benefits of beta blockers in patients with heart failure with reduced ejection fraction (HFrEF),^17–19^ there are few reports describing the effect of beta blockers on patients undergoing TAVR. An observational study reported that the absence of beta blockers was an independent predictive factor of death following TAVR in patients with a history of chest irradiation.^20^

Two remaining and frequent complications of TAVR are PVL and pacemaker implantation. Administration of beta blockers is considered harmful in patients with AR because of a longer diastolic period. Additionally, beta blockers suppress atrioventricular conduction. Therefore, there was concern that beta-blocker administration would increase the risk and impact of these complications. However, there was no significant difference in PVL degree and new permanent pacemaker implantation rate between patients with and without beta-blocker administration in this study. On the contrary, Younis *et al* reported that beta-blocker discontinuation was associated with an increased risk of high degree AV block or AF among patients with TAVR.^21^

In this study, beta blockers were associated with a lowered non-cardiovascular mortality in patients with COPD. It was assumed that beta blockers were contraindicated in patients with COPD because of potential exacerbation. Recently, beta-blocker use in patients with COPD was associated with decrease in all-cause mortality and exacerbation rate.^22^ However, these results should not be overstated and are beyond the scope of this study.

Blessberger *et al* reported that there was no clear evidence of the prevention of beta blockers on congestive heart failure after cardiac surgery.^23^ However, they also reported that perioperative beta-blocker administration reduces the risk of ventricular and supraventricular arrhythmias. These findings may have led to a lower incidence of in-hospital congestive heart failure among patients treated with beta blockers than among those not treated with beta blockers.

All specific groups identified in this study are at high risk of heart failure or cardiovascular event. Patients undergoing TAVR with a history of CABG, presence of PAD, high BNP levels or LVEF <50% had high mortality rates.^24–27^ The possible benefits of beta blockers for high-risk patients with heart failure or cardiovascular events could be a potential explanation for the association of a low cardiovascular mortality with beta-blocker administration in these groups of patients. The efficacy of beta blockers in the treatment of ischaemic heart disease and heart failure has been established. Patients with PAD have a high possibility of polyvascular disease, such as CAD and cerebrovascular disease. In addition, Satiroglu *et al* reported that the lesion of CAD in patients with PAD was often more severe than that in patients without PAD.^28^ For that reason, beta blockers may be effective on patients with PAD undergoing TAVR. Some prior studies have shown that beta-blocker therapy was associated with a low risk of mortality in these patients.^17–19 29 30^

In this study, there was no association between the presence and absence of beta-blocker administration in cardiovascular mortality in patients with pre-LVEF <50% or ≥50%, although beta-blocker administration was associated with a lower cardiovascular mortality among patients with post-LVEF <50%. This result may suggest that beta blockers are effective in patients with LVEF <50% even after severe AS improvement by TAVR. Patients with LVEF <50% included those with HFrEF and with mid-range HFEF (HFmEF). A recent meta-analysis showed that beta blockers reduced cardiovascular mortality in patients with HFrEF and HFmEF.^31^

In this study, we found that beta blockers were not associated with the risk of increased complications and non-cardiovascular mortality. Additionally, we identified the association with beta blockers and clinical outcomes. The optimal medical therapy for patients who undergo TAVR would be important because the indications for TAVR are expanding and the number of these patients is increasing. However, this study could not identify the doses and types of beta blockers. Prospective studies are needed to evaluate these issues.

### Study limitations

This study has several limitations. First, it was a non-randomised, retrospective study using data from a prospective multicentre cohort registry including a limited number of patients; however, this cohort is representative of real-world practice. Further studies with a larger number of patients are ongoing. Second, the definition of in-hospital congestive heart failure is unique to this registry. Additionally, no information was available on the amounts, types of infusion or drugs used during the procedure that may affect the onset of congestive heart failure. The result of low incidence of in-hospital congestive heart failure is beyond the scope of the effect of beta blockers because the definition includes mechanical support for heart failure, such as IABP and ECMO. In addition, inotropic agents and mechanical support were also used not only for heart failure but also for the cardiogenic shock management. In-hospital congestive heart failure did not include the cases in which inotropic agents or mechanical support was used for other than heart failure. However, the reasons for the use of inotropic agents or mechanical support were site reported from the participating institutions. The diagnosis of heart failure was left to the discretion of the clinicians in each participating institution. Third, no information regarding the dose or period of administration, time schedule or type of beta blockers was included. There was not enough information of medication status at follow-up. In randomised controlled trials, carvedilol, bisoprolol or metoprolol succinate reduce all-cause mortality in patients with HFrEF.^17–19^ Additionally, the effect of beta blockers is dose dependent.^32^ Fourth, there was no information on the blood pressure and heart rate of patients involved in this study. A low heart rate is associated with good prognosis in patients with heart failure,^32^ and it is not recommended to use beta blockers in patients with hypotension or bradycardia. Lastly, there was a possibility that some of the significant results were due to the multiple testing. In addition, the number of the events of secondary outcomes and the event in the subgroups was relatively small. The statistical power may be limited. Therefore, more meticulous studies including larger patients that consider these should be our future targets.

### Conclusions

Preprocedural beta-blocker administration for patients undergoing TAVR was not associated with 2-year cardiovascular and non-cardiovascular mortality in overall, but was associated with a lower risk of 2-year cardiovascular mortality only in patients with a history of CABG, presence of PAD, BNP ≥400 pg/mL and post-LVEF <50%. These patients may be better prescribed beta blockers. The findings need to be confirmed by randomised trials.
