# Supplementary material for: Impact of beta blockers on patients undergoing transcatheter aortic valve replacement: the OCEAN-TAVI registry
Source: Open Heart. 2020 Jul 7;7(2):e001269. doi: 10.1136/openhrt-2020-001269 (PMC7342827; doi:10.1136/openhrt-2020-001269)

Supplementary Table 1. The percentage of missing data for baseline variables.

Variables	Percentage of missing data (%)
BNP	12.8
Indexed stroke volume	8.7
SOV mean diameter	3
Left ventricular end-systolic diameter	0.42
Left atrial diameter	0.42
Annulus area	0.15
Left ventricular end-diastolic diameter	0.15
AVA	0.11
Mean pressure gradient	0.11
Euro Ⅱ score	0.11
LVEF	0.07
Na	0.04
Peak velocity	0.04
AR	0.04

Abbreviations: AR = aortic regurgitation; AVA = aortic valve area; BNP = brain natriuretic peptide; LVEF = left ventricular ejection fraction; SOV = sinus of Valsalva.

Supplementary Table 2. Baseline variables after propensity score matching.

	Beta-blocker (+) n=779	Beta-blocker (-) n=779	P value	SMD
Age, yrs	85(82-88)	85(81-88)	0.57	0.029
Male	239(30.7)	232(29.8)	0.7	0.020
Body mass index, kg/m^2	21.9(19.4-24.4)	22.2(19.6-24.4)	0.73	0.018
Body surface area, m^2	1.40(1.30-1.52)	1.40(1.30-1.53)	0.96	0.003
NYHA 3 or 4	419(53.8)	424(54.4)	0.8	0.013
Hypertension	613(78.7)	619(79.5)	0.71	0.019
Dyslipidemia	333(42.7)	335(43.0)	0.92	0.005
Diabetes mellitus	174(22.3)	192(24.6)	0.28	0.055
Chronic kidney disease	568(72.9)	562(72.1)	0.73	0.017
Previous ischemic stroke	72(9.2)	78(10.0)	0.61	0.026
Previous hemorrhagic stroke	3(0.4)	4(0.5)	0.71	0.019
COPD	117(15.0)	135(17.3)	0.22	0.063
Peripheral artery disease	126(16.2)	124(15.9)	0.89	0.007
Coronary artery disease	306(39.3)	303(38.9)	0.88	0.008
Previous CABG	56(7.2)	63(8.1)	0.5	0.034
Atrial fibrillation	183(23.5)	179(23.0)	0.81	0.012
Permanent pacemaker	56(7.2)	60(7.7)	0.7	0.034
Liver disease	23(3.0)	23(3.0)	1	<0.001
Active cancer	39(5.0)	37(4.7)	0.81	0.012
CFS			0.92	0.021
1-4	590(75.7)	589(75.6)		
5,6	163(20.9)	161(20.7)		
7,8	26(3.3)	29(3.7)		
STS score, %	6.81(4.74-9.96)	6.60(4.70-9.91)	0.6	0.007
Logistic EuroScore, %	13.1(8.68-21.2)	13.1(8.45-21.4)	0.87	0.006
Euro Ⅱ score, %	3.87(2.51-6.03)	3.88(2.48-6.38)	0.91	0.001
Medication				
RAS inhibitors	431(55.3)	426(54.7)	0.74	0.013
Ca blockers	346(44.4)	347(44.5)	0.96	<0.001

Digoxin	22(2.8)	16(2.1)	0.32	0.050
Any diuretic therapy	486(62.4)	496(63.7)	0.6	0.027
Statin	333(42.7)	345(44.3)	0.54	0.031
Laboratory				
Na, mEq/l	140(138-142)	140(138-142)	0.67	0.012
Hb, g/dl	11.1(10.1-12.3)	11.2(10.1-12.4)	0.93	0.010
eGFR, ml/min/1.73m^2	48.4(36.6-61.0)	49.0(36.7-61.2)	0.6	0.009
Albumin, g/dl	3.80(3.40-4.00)	3.80(3.50-4.10)	0.71	0.010
Albumin < 3.5g/dl	196(25.2)	192(24.6)	0.82	0.012
Brain natriuretic peptide, pg/ml	332(153-620)	292(123-626)	0.14	0.023
Brain natriuretic peptide ≥ 400 pg/ml	266(41.5)	280(41.9)	0.88	0.008
Pre-procedural echocardiographic data/Computed tomographic data				
Aortic valve are, cm2	0.61(0.50-0.73)	0.62(0.50-0.73)	0.56	0.018
Peak velocity, m/s	4.47(4.01-5.00)	4.44(4.00-5.00)	0.95	0.001
Mean pressure gradient, mmHg	46.0(37.0-58.9)	46.9(37.0-59.0)	0.8	<0.001
LV end-diastolic diameter, mm	43.5(39.2-48.2)	44.0(40.0-49.0)	0.43	0.018
LV end-systolic diameter, mm	28.0(24.5-33.8)	28.3(25.0-33.5)	0.61	0.012
Left atrial diameter, mm	42.5(37.4-47.0)	41.5(38.0-46.0)	0.29	0.002
LVEF, %	61.2(52.0-67.0)	61.0(51.2-67.0)	0.63	0.028
LVEF <50%	164(21.1)	168(21.6)	0.81	0.013
Indexed stroke volume, ml/m^2	43.2(34.4-53.0)	43.6(34.8-52.4)	0.89	0.013
Indexed stroke volume <35ml/m^2	174(26.6)	165(25.9)	0.77	0.016
AR ≥moderate	80(10.3)	92(11.8)	0.33	0.049
MR ≥moderate	89(11.4)	98(12.6)	0.48	0.036
Bicuspid	20(2.6)	15(1.9)	0.39	0.043
Annulus area, mm^2	390(347-442)	391(350-438)	0.88	0.001
SOV mean diameter, mm	29.4(27.7-31.4)	29.4(27.5-31.6)	0.74	0.008
Procedural data				
Transfemoral approach	636(81.6)	636(81.6)	1	<0.001
Elective	733(94.1)	737(94.6)	0.66	0.022
Local anesthesia	183(23.5)	179(23.0)	0.81	0.012

Abbreviations: AR = aortic regurgitation; CABG = coronary artery bypass grafting;

COPD = chronic obstructive pulmonary disease; eGFR = estimated glomerular filtration rate; LV = left ventricular; LVEF = left ventricular ejection fraction; MR = mitral regurgitation; NYHA = New York Heart Association; RAS = renin-angiotensin system; SMD = standardized mean difference; SOV = Sinus of Valsalva; STS = Society of Thoracic Surgeons Predicted Risk of Mortality.

Supplementary Figure 1. Subgroup analysis for cardiovascular mortality in the subset of PPM and PVL.

Forrest plot representing the hazard ratios of cardiovascular mortality in patients with beta-blocker administration compared to patients without, stratified by PPM, PVL.

Abbreviations: PPM = prosthesis-patient mismatch; PVL = paravalvular leak.

Supplementary Figure 1

Subgroup	No. of patients	No. of event/total no. of patients Beta-blocker(-)	Beta-blocker(+)	Hazard ratio(95% CI)	p for interaction
Overall	1558	83/779	71/779	0.84(0.61-1.16)	
PPM-	1336	39/669	34/667	0.87(0.55-1.438)	p=0.15
PPM+	179	3/85	8/94	2.44(0.65-9.18)	
PVL					
none	267	9/127	4/140	0.38(0.12-1.22)	p=0.34
trivial	743	17/377	26/366	1.63(0.89-3.01)	
mild	511	20/259	12/252	0.59(0.29-1.21)	
moderate	31	2/11	1/20	0.25(0.02-2.75)	
severe	1	0	1/1	N/A	

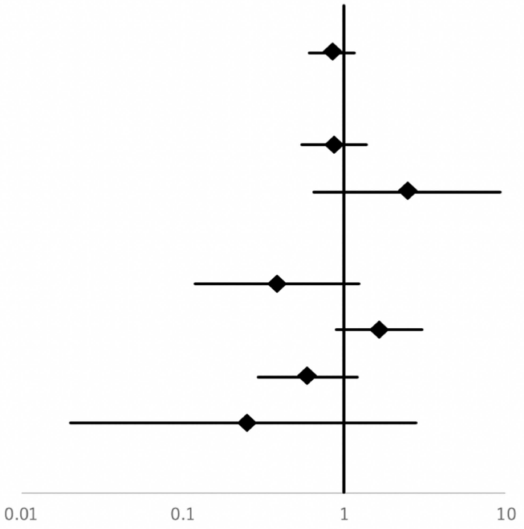

Supplement: Supplementary data [file openhrt-2020-001269supp001.pdf]
